# Can fisheries bioenergetics modelling refine spatially explicit assessments of climate change vulnerability?

**DOI:** 10.1093/conphys/coac035

**Published:** 2022-07-02

**Authors:** Matthew J Troia, Joshuah S Perkin

**Affiliations:** Department of Integrative Biology, University of Texas at San Antonio, San Antonio, TX 78249, USA; Department of Ecology and Conservation Biology, Texas A&M University, College Station, TX 77843, USA

## Abstract

Rising water temperature under climate change is affecting the physiology, population dynamics and geographic distribution of freshwater taxa. We propose a novel application of individual-based bioenergetics modelling (BEM) to assess the physiological impacts of warming on freshwater fishes across broad spatial extents. We test this approach using the Guadalupe bass (*Micropterus treculii*), a species of conservation and recreational significance that is endemic to central TX, USA. We projected historical-to-future changes (middle 20th century to end of 21st century) in daily bioenergetics of individual fish across 7872 stream reaches and compared this output to changes in reach occupancy derived from traditional species distribution modelling (SDM). SDMs project an 8.7% to 52.1% decrease in reach occupancy, depending on model parameterizations and climate change scenarios. Persistence is projected in the central Edwards Plateau region, whereas extirpations are projected for the warmer southeastern region. BEM projected a median 79.3% and 143.2% increase in somatic growth of age-1 Guadalupe bass across historically occupied reaches under moderate and severe climate change scenarios, respectively. Higher end-of-year body size under future climate was caused by a longer growing season. Future scenarios exploring suppressed or enhanced prey consumption suggest that small changes in prey availability will have relatively greater effects on growth than forecasted changes in temperature. Projected growth was geographically discordant with SDM-based habitat suitability, suggesting that SDMs do not accurately reflect fundamental thermal niche dimensions. Our assessment suggests that for locations where the species persists, Guadalupe bass may benefit from warming, although realized consumption gains will depend on seasonal, spatially varying changes in prey availability and other biotic and abiotic factors. More generally, we demonstrate that uniting species-specific BEM with spatially explicit climate change projections can elucidate the physiological impacts of climate change—including seasonal variation—on freshwater fishes across broad geographic extents to complement traditional SDM.

## Teaser

We used bioenergetics models to project the physiological impacts of climate warming across the geographic range of Guadalupe bass, *Micropterus treculii*, and compared these projections to changes in habitat suitability from traditional species distribution models. This comparison revealed complementary insights that can inform conservation of freshwater fishes experiencing climate change.

## Introduction

Climate change poses a significant threat to global biodiversity in the 21st century. Conservation planners need vulnerability assessments to identify and prioritize which species and regions are most in need of conservation action. Many of these assessments implement correlative species distribution modelling (SDM; *sensu*  Elith and Leathwick, 2009) because the input data—georeferenced occurrence records and geographic information system (GIS)-based climate layers—are profuse and easily accessible (Anderson *et al.*, 2016; Fick and Hijmans, 2017). This approach facilitates the assessment of many species across broad geographic areas. For example, Crossman *et al.* (2012) used the magnitude of SDM-projected distributional shifts between historical and future climates to characterize vulnerability of 171 southern Australian plant species. In a similar study of 270 Australian Odonate dragonflies and damselflies, Bush *et al.* (2014) identified taxa that are most vulnerable to climate change and identified priority regions for conservation actions such as regulating water extraction and protecting refugia habitats. Numerous other assessments have been performed for a variety of regions and taxa, including freshwater fishes (Herrera-R *et al.*, 2020; Perkin *et al*., 2021; Troia *et al*., 2019). These SDM-based assessments are invaluable to conservation planners because they incorporate many species in the assessment, are spatially contiguous and span broad geographic extents.

A limitation of SDM-based assessments is that they do not explicitly assess physiological impacts of climate change. This can lead to uncertain and inaccurate vulnerability assessments because the realized climatic niche in the present may not reflect the fundamental climatic niche (Kearney and Porter, 2009; Sax *et al*., 2013). Recent assessments are beginning to estimate the physiological impacts of climate change across broad spatial extents. For example, Comte and Olden (2017) used laboratory-derived upper thermal tolerances of fishes and global temperature projections to assess global patterns of vulnerability among freshwater and marine fishes. This assessment revealed high warming exposure for temperate freshwater faunas versus high physiological sensitivity for temperate marine faunas. Similarly, Dillon *et al*. (2010) found that metabolic sensitivity of terrestrial ectotherms is higher for tropical taxa compared with temperate and polar taxa, suggesting that physiological impacts of climate warming will be greater in the tropics despite relatively moderate rates of warming. These physiology-based assessments are important because they can reveal causal links between specific climatic conditions and the performance of individuals and populations. For example, Briscoe *et al*. (2016) showed that timing of rainfall events relative to extreme heat events limit survival of koalas at their contemporary range edges and will likely drive shifts in these range edges under future climate.

Freshwater fishes are diverse, imperilled and in need of vulnerability assessments and conservation action (Strayer and Dudgeon, 2010; Vörösmarty *et al.*, 2010). Bioenergetics modelling (BEM) has been used in fisheries science since the 1970s to inform the management of recreational and commercial fish stocks (Augustine and Kooijman, 2019; Deslauriers *et al*., 2017). The ‘Wisconsin model’ (*sensu*  Deslauriers *et al.*, 2017) uses a species-specific energy balance equation to estimate energy gains—via consumption of prey, and energy losses, via metabolism, egestion and excretion—to estimate daily energy budgets of individuals. These energy balance equations are based on intrinsic physiological and behavioural traits of the species and on extrinsic environmental conditions. Prolonged periods of bioenergetic deficits lead to mortality, whereas bioenergetic surpluses are directed towards somatic growth and reproduction (Garvey *et al.*, 2004; Lawrence *et al*., 2015). BEM provides a physiology-based approach to assess climate change vulnerability of fishes that go beyond assessments based on physiological thermal limits (e.g. Comte and Olden, 2017; Deutsch *et al.*, 2008; Feeley and Silman, 2010). Pease and Paukert (2014) used BEM to estimate prey consumption and growth potentials for four populations of smallmouth bass (*Micropterus dolomieu*) under historical and future thermal regimes. Nevertheless, there are few such applications of BEM. It is surprising that BEM has not been used for broader-scale climate change vulnerability assessments given (i) the accumulation of species for which BEM are parameterized (i.e. at least 70 species for the Wisconsin model; Deslauriers *et al.*, 2017); (ii) the accessibility of GIS data layers describing environmental conditions in freshwater habitats (Hill *et al*., 2016); and (iii) conceptual development of BEM for conservation of rare species (Petersen *et al*., 2008).

**Figure 1:**
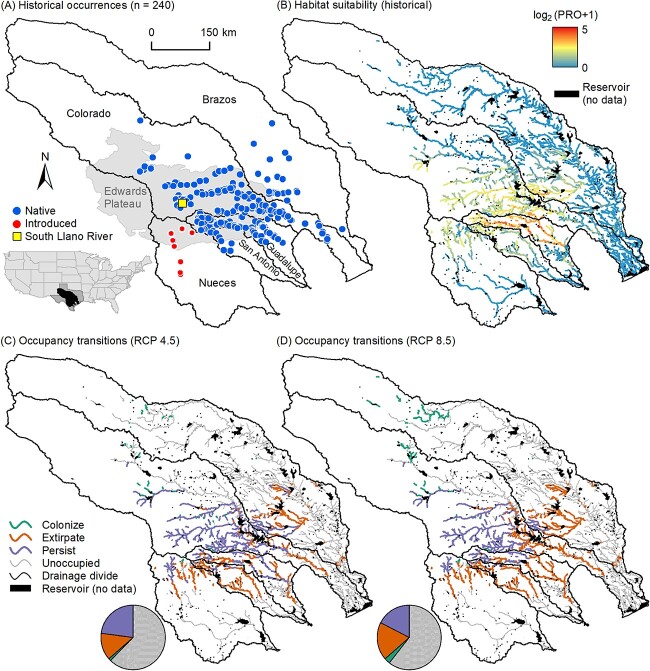
Distribution of Guadalupe bass in central Texas. (**A**) Distribution of IchthyMaps occurrence records, (**B**) SDM-projected habitat suitability and SDM-projected occupancy transitions between historical and future climate scenarios assuming (**C**) moderate and (**D**) high emissions scenarios. Pie charts show proportion of 7872 reaches in each occupancy transition, and the yellow square denoting the South Llano River is the location from which growth data were obtained. Panels (C) and (D) illustrate projections 2 and 5 from Table 1, respectively.

Here, we present an assessment of climate change vulnerability for the Guadalupe bass (*Micropterus treculii*)—a fish endemic to the Edwards Plateau region of central TX, USA (Fig. 1A). The small geographic range, only 20 000 to 200 000 km^2^ (NatureServe, 2010), and the ongoing threat of hybridization with non-native smallmouth bass (*M. dolomieu*; Bean *et al.*, 2013) make Guadalupe bass a species of conservation concern (Hubbs *et al*., 2008). Guadalupe bass are also popular among recreational anglers, meaning the persistence of robust populations is of cultural and economic importance for the region (Thomas, 2015). Still, vulnerability of Guadalupe bass to 21st century climate change remains unknown. To assess climate change vulnerability, we used BEM to project changes in bioenergetic budgets of individuals and correlative SDMs to project changes in reach occupancy under historical and end of 21st century climate scenarios. Because BEM parameters do not yet exist for Guadalupe bass, we compiled parameters of largemouth bass (*Micropterus salmoides*) and used a novel parameter resampling algorithm to develop and validate an ensemble of BEMs. SDMs were parameterized with open-source occurrence records and landscape and climatic covariates using the Maximum Entropy algorithm (Phillips *et al.*, 2004; Vollering *et al*., 2019). Both models were projected to 7872 perennial confluence-to-confluence stream reaches within the Guadalupe bass range. By comparing occurrence-based and physiology-based approaches using this broad-scale, spatially explicit framework of SDM, we provide novel insight into how climate change will (i) affect multiple levels of the biological hierarchy and (ii) vary across Guadalupe bass geographic range, including where vulnerability (or benefits) will be greatest. Implementing BEM on a daily time step also reveals seasonal variation in bioenergetic budgets, providing insight into another dimension of vulnerability—seasonal changes in physiological performance.

## Materials and methods

### Study system

The Guadalupe bass is native to the Brazos, Colorado, Guadalupe and San Antonio river basins and is introduced to the Nueces river basin of TX, USA (Fig. 1A). Streams of these basins flow generally from northwest to southeast. Most of these basins drain the High Plains in the west, Edwards Plateau in the center and the Coastal Plain in the east. Precipitation declines markedly from 1360 mm per year in the east to 345 mm per year in the west and mean annual air temperature ranges from 13.5°C in the northwest to 22.1°C in the south. We implemented SDMs and BEM for all perennial, confluence-to-confluence stream reaches (hereafter ‘reaches’) within the five basins. We acquired reaches as a polyline shapefile from the National Stream Internet flowline layer (Nagel *et al.*, 2017) and retained perennial reaches (NHD FCode = 46 006), yielding 7872 reaches.

### Species distribution modelling

We used SDM to map the habitat suitability of Guadalupe bass to all 7872 reaches in the study region. We extracted records of Guadalupe bass occurrence (*n* = 240) between 1950 and 1980 from the IchthyMaps dataset (Frimpong *et al*., 2016) (Fig. 1A). We acquired GIS-derived predictor variables describing hydrographic, geological and soil characteristics (hereafter ‘landscape’ variables) for the 7872 reaches from the StreamCat dataset (Hill *et al.*, 2016). We acquired averages of monthly precipitation totals and monthly air temperature minima, means and maxima (hereafter ‘climate’ variables) for all reaches between 1960 and 1990 using the ClimateNA program (Wang *et al*., 2016). Although air temperature and precipitation are not direct measures of parallel in-stream conditions (water temperature and flow, respectively), these climatic conditions are effective surrogate covariates for modelling habitat suitabilities of stream-dwelling fishes (McGarvey *et al.*, 2018). We checked variable pairs for correlations (Pearson’s *r*) and retained only uncorrelated variables (|*r*| ≤ 0.7) known to affect in-stream habitat suitability of stream fishes (Huang and Frimpong, 2015) (Supplementary Table S1).

We fit SDMs with the Maximum Entropy algorithm using the MIAmaxent library in R (Vollering *et al.*, 2019). This model was used to project habitat suitability to all 7872 reaches under seven different projections, each representing a different combination of climate scenarios (Table 1). For projections to future climates (projections 2–7), we used scenarios of moderate (Representative Concentration Pathway; hereafter ‘RCP 4.5’) and high (RCP 8.5) greenhouse gas emissions derived from an ensemble of 15 general circulation models (Wang *et al.*, 2016). Because we were interested in exploring effects of temperature and precipitation changes independently of one another—and comparing temperature-only effects to BEM projections—we performed projections in which both temperature and precipitation change (projections 2 and 5), only temperature changes (3 and 6) or only precipitation changes (4 and 7). We used the log_2_ of the probability ratio output (hereafter ‘PRO’) as habitat suitability and interpreted reaches with values ≥1 as being occupied following Vollering *et al.* (2019). We defined four historical to future projection states: persist (occupied to occupied), colonize (unoccupied to occupied), extirpate (occupied to unoccupied) and unoccupied (unoccupied to unoccupied). Our definition of ‘occupied’ is based strictly on habitat suitability of the reach and does not account for dispersal limitation, particularly as it relates to the ‘colonize’ state.

**Table 1:** Summary of SDM projection conditions and results

	Projection conditions				Projection results				
Projection	Time period	Emissions scenario	∆ T^a^	∆ P^a^	Occupied^b^	Unoccupied^b^	Colonize^b^	Extirpate^b^	% change occupancy
1	1960–1990	Historical			2871	5001	n/a	n/a	n/a
2	2071–2100	RCP 4.5	✓	✓	1806	4909	92	1065	−37.1%
3	2071–2100	RCP 4.5	✓		2036	4913	88	835	−29.1%
4	2071–2100	RCP 4.5		✓	2496	4843	158	375	−13.1%
5	2071–2100	RCP 8.5	✓	✓	1375	4771	230	1496	−52.1%
6	2071–2100	RCP 8.5	✓		1645	4772	229	1226	−42.7%
7	2071–2100	RCP 8.5		✓	2622	4884	117	249	−8.7%

^a^Check mark indicates future climate variables were used for SDM projection, whereas no check mark indicates historical climate was used.

b Number of reaches out of 7872 perennial reaches.

We evaluated model performance by splitting records into a 50% training set (*n* = 120), fitting the model and computing the area under the curve (AUC) of the receiver operator characteristic on the remaining records (testing set; *n* = 120 records). Because the IchthyMaps dataset does not include putative absence records, we selected a random sample (equal to the number of records in the testing set; *n* = 120) of reaches in which a congener (*Micropterus spp.*) was present in the IchthyMaps dataset and Guadalupe bass was not present. The presence of an ecologically similar species and not the target species increases the likelihood of selecting true absences compared to selecting random reaches (with or without) IchthyMaps records (Huang and Frimpong, 2015; Mateo *et al*., 2010). This independent cross-validation procedure was repeated 10 times, each time selecting different subsets of records for training and testing. The mean AUC of these 10 repetitions was computed and used to evaluate model performance. We evaluated variable performance by removing each variable, in turn, and computing the percent reduction in AUC relative to the model with all variables. To evaluate the predictive capability of landscape versus climate variables, we evaluated model performance of models fit with (i) landscape and climate variables, (ii) landscape variables only and (iii) climate variables only.

### BEM—model descriptions

We used BEM to map somatic growth potential (hereafter ‘growth’) of Guadalupe bass to all 7872 reaches in the study region. This is the Wisconsin bioenergetics model, which follows the equation from Hanson *et al*. (1997):

(1) \begin{equation*} G=C-\left(R+ SDA+E+U\right) \end{equation*}

where *G* is energy gained via growth, *C* is energy gained via consumption, *R* is energy lost via respiration, SDA is energy lost via specific dynamic action (the energetic cost of digestion), *E* is energy lost via egestion and *U* is energy lost via excretion. Mass dependence of respiration and consumption are modelled as power functions of the form:

(2) \begin{equation*} R={R}_A\times {M}^{R_B}\times f\left({T}_R\right)\times {R}_{ACT} \end{equation*}

(3) \begin{equation*} C={C}_A\times {M}^{C_B}\times f\left({T}_C\right)\times {C}_p, \end{equation*}

where *R* and *C* are mass-specific routine metabolism measured in grammes of O_2_ consumed per gramme of body mass and maximum consumption as grammes of prey consumed per gramme of body mass, respectively; *M* is mass; *R_A_* and *C_A_* are slopes; and *R_B_* and *C_B_* are *y*-intercepts. *f(T_R_)* and *f(T_C_)* are temperature dependence of respiration and consumption, represented by Equations 4 and 5, respectively. BEM also require inputs of energy density, the proportion of maximum consumption at which an individual feeds (*C_P_*), and the activity multiplier of respiration (*R_ACT_*) that takes into account the metabolic costs of activity beyond routine metabolic rate (e.g. searching for and pursuing prey). We converted O_2_ consumption to joules using the oxycalorific coefficient, 13 560 J/g O_2_ (Hanson *et al.*, 1997) and grammes of prey consumed to joules using the median energy density reported for larval fish, the presumed prey of age-1 Guadalupe bass (3698 J/g wet mass; Henderson and Ward, 1978). The temperature dependence equation for respiration follows the exponential form presented by Hanson *et al.* (1997):

(4) \begin{equation*} f\left({T}_R\right)={e}^{R_Q\times T}, \end{equation*}

where *f(T_R_)* is mass-specific routine respiration, *R_Q_* is the slope of the respiration function at low water temperatures and *T* is temperature. The temperature dependence equation for consumption follows the monotonic form presented by Hanson *et al.* (1997) after Kitchell *et al*. (1977):

(5) \begin{equation*} f\left({T}_C\right)={V}^X\times {e}^{\left(X\times \left(1-V\right)\right)}, \end{equation*}

where

$$ V=\left( CTM-T\right)/\left( CTM- CTO\right) $$

$$ X=\left({Z}^2\times {\left(1+{\left(1+40/Y\right)}^{0.5}\right)}^2\right)/400 $$

$$ Z=\ln \left({C}_Q\right)\times \left( CTM- CTO\right) $$

$$ Y=\ln \Big({C}_Q\left( CTM- CTO+2\right), $$

where *f(T_C_)* is mass-specific consumption, *CTM* is the temperature above which feeding ceases, *CTO* is laboratory temperature preferendum and C*_Q_* approximates a *Q_10_* (i.e. the rate at which consumption increases at low temperature). We assumed egestion and excretion to be constant proportions of consumption and specific dynamic action to be a constant proportion of respiration (Hanson *et al.*, 1997).

Because laboratory measurements of the mass-dependence and temperature-dependence of consumption and respiration as well as energy density have not been published for Guadalupe bass (Deslauriers *et al.*, 2017), we borrowed these BEM parameters from largemouth bass (Supplementary Table S2). Largemouth bass is the most recent common ancestor of Guadalupe bass, having diverged approximately 5.2 million years ago (Near *et al*., 2003), and occupies a climatic envelope that contains Guadalupe bass (NatureServe, 2010). As such, largemouth bass is the best surrogate species currently available for modelling bioenergetics budgets of Guadalupe bass (Petersen *et al.*, 2008). Mass-dependent respiration and consumption were modelled as power functions (Equations 2 and 3). Temperature-dependent respiration was modelled as an exponential function (Equation 4) and temperature-dependent consumption was modelled as a monotonic function (Equation 5). Because it is unlikely that BEM parameters of Guadalupe bass are precisely equivalent to largemouth bass, we performed a parameter resampling procedure to generate additional parameter sets (hereafter ‘synthetic parameter sets’). For each parameter, we sampled from a uniform distribution bounded by a minimum value 5% lower than the largemouth bass parameter value and a maximum value 5% higher than the largemouth bass parameter value. These bounds generated parameter values that are within the range of parameter values observed for other fish species (Deslauriers *et al.*, 2017), and therefore are biologically realistic. We used this procedure to generate 500 synthetic parameter sets.

### BEM—validation

We validated BEM using empirical growth of a Guadalupe bass population in the South Llano River. We estimated age-specific growth using back-calculated length-at-age measurements of scales taken from fish spawned between 2005 and 2011 (Groeschel-Taylor *et al*., 2019). Lengths-at-age were converted to masses-at-age using von Bertalanffy equations of Guadalupe bass (Groeschel-Taylor *et al.*, 2019). We used BEM to project growth beginning on Julian day 1 and ending on Julian day 365. These projections require four inputs: (i) initial fish mass, (ii) proportion of maximum consumption at which an individual feeds (*C_P_*), (iii) the activity multiplier of respiration (*R_ACT_*) and (iv) mean daily water temperature during the projection period. Fish mass was assumed to be 6.4 g for age-1 fish (Groeschel-Taylor *et al.*, 2019), *C_P_* to be 0.5 and *R_ACT_* to be 1.0. We estimated mean daily water temperatures for all 17 reaches within the South Llano River study area of Groeschel-Taylor *et al.* (2019) using a two-step approach. First, we acquired monthly mean air temperatures at each reach from the ClimateNA program (Wang *et al.*, 2016) for the years during which growth was measured (2005 through 2011). We converted air temperature to water temperature using sigmodal air-water temperature relationships derived by Punzet *et al*. (2012) for the warm-temperate Köppen–Geiger climate zone. Second, we fit a sine function to the annual temperature cycle at each reach, separately, using the monthly water temperatures and assuming these monthly values correspond to the middle day of each month (i.e. Julian days 15, 46, 74, 105, 135, 166, 196, 227, 258, 288, 319 and 349). We then used these sine functions to interpolate water temperature to all 365 Julian days at the 17 reaches.

We simulated growth for age-1 individuals using each of the 500 synthetic parameter sets, and identified those that projected end-of-*growing-season* simulated length within ±2.5% of end-of-*year* empirical length and retained these BEM for the climate change vulnerability assessment described below. We used simulated length at the end-of-growing-season (i.e. last Julian day with a daily bioenergetics surplus) because mass declines during the remaining days of the calendar year whereas length does not. Hence, using simulated end-of-year mass and converting it to length would underestimate actual length gained during the calendar year.

### BEM—projecting bioenergetics to reaches

We used only the validated parameter sets to project growth of age-1 Guadalupe bass for all 7872 reaches assuming climate under the historical time period and four future scenarios: (i) RCP 4.5 emissions scenario with baseline consumption (*C_P_* = 0.5), (ii) RCP 8.5 with baseline consumption (*C_P_* = 0.5), (iii) RCP 4.5 scenario with consumption suppressed by 10% (*C_P_* = 0.45) and (iv) RCP 4.5 scenario with consumption enhanced by 10% (*C_P_* = 0.55). We assumed fish mass was 6.4 g on Julian day 1 and projected growth through Julian day 365. We estimated mean daily water temperatures for all 7872 reaches using the same two-step approach described above. We used these simulated growth trajectories to estimate end-of-year mass. We also used daily bioenergetics budgets to explore the duration of growing season, which we defined as the number of consecutive days of bioenergetics surplus. Projections of daily bioenergetics budget and end-of-year mass were expressed as the mean of the validated parameter sets. Uncertainty in these projections sets was expressed as the coefficient of variation among validated parameter sets. All BEM were developed and projected using original scripts in R.

### Statistical analyses

We used generalized additive models (GAM) to evaluate the spatial (i.e. among reaches) concordance between BEM and SDM projections. We fit three sets of models to test three statistical hypotheses: (i) historical end-of year body size is positively correlated with historical habitat suitability (Hypothesis 1); (ii) historical end-of year body size is positively correlated with change in habitat suitability between historical and future time periods (Hypothesis 2); and (iii) change in end-of-year body size between historical and future time periods is positively correlated with change in habitat suitability between historical and future time periods (Hypothesis 3). Confirmation of Hypothesis 1 would support the biological hypothesis that low habitat suitability limits growth capacity at range margins, whereas high habitat suitability facilitates greater growth capacity within the core of the range. Confirmation of Hypothesis 2 would support the biological hypothesis that historical habitat suitability will maintain conditions suitable for growth capacity in the future. Confirmation of Hypothesis 3 would support the biological hypothesis that historical to future reduction in growth capacity will be caused by the reduction in habitat suitability, whereas increased habitat suitability will facilitate increased growth capacity. Alternatively, no relationship or a negative relationship would indicate that historical spatial variation in habitat suitability or historical to future change in habitat suitability is associated with other aspects of individual performance (e.g. behaviour, reproduction, recruitment, etc.).

For both model sets, SDM-based habitat suitability [i.e. log_2_(PRO+1)] was the predictor variable and BEM-based growth was the response variable. Both model sets also included alternative models with ecoregion (Supplementary Fig. S1) fit as a factor because inspection of mapped projections revealed regional differences in BEM and SDM outputs. Models were fit using restricted maximum likelihood in the mgcv library in R (Wood and Scheipl, 2014). Model significance was assessed using an *F*-test at *P* < 0.05 and performance was assessed using deviance explained.

## Results

### Species distribution models

SDMs performed moderately well when fit with landscape variables only (mean AUC = 0.78) or with landscape variables and climate variables (0.79), but poorly when only climate variables were used (0.68). The most important predictor variable was baseflow index (percent change in AUC = −2.01%), followed by January minimum temperature (−1.13%) and soil erodibility factor (−1.09%) (Supplementary Table S1). These relatively small percent changes in AUC suggest that the environmental niche space of GB is not strongly defined by any single environmental gradient.

SDMs projected the highest historical habitat suitability for streams within the Edwards Plateau, particularly those within the Guadalupe River basin, as well as the mainstem Guadalupe and San Marcos Rivers to the southeast of the Edwards Plateau (Fig. 1B). Projected reach occupancy under historical climate was 2871 reaches, constituting 36.5% of the 7872 perennial reaches within the five-basin range of Guadalupe bass. Occupancy is projected to decrease (−8.7 to −52.1% change) between the historical and future time periods (Table 1). SDM projections that assume only temperature changes (Projections 3 and 6) project more reach extirpations (−29.1% to −42.7% change) than projections that assume only precipitation changes (Projections 4 and 7; −8.7% to −13.1% change), suggesting that historical distributions are limited more by temperature than by precipitation. Extirpations are projected for reaches to the south and east of the Edwards Plateau, whereas relatively few colonizations (Table 1) are projected for reaches to the north of the Edwards Plateau (Fig. 1C, D). This spatial pattern of occupancy transition is similar for projections that assume only temperature changes (Supplementary Fig. S2A, B). For projections assuming only precipitation changes, extirpations are limited to reaches in the arid southwestern region (Supplementary Fig. S2C, D).

### Bioenergetics models

**Figure 2:**
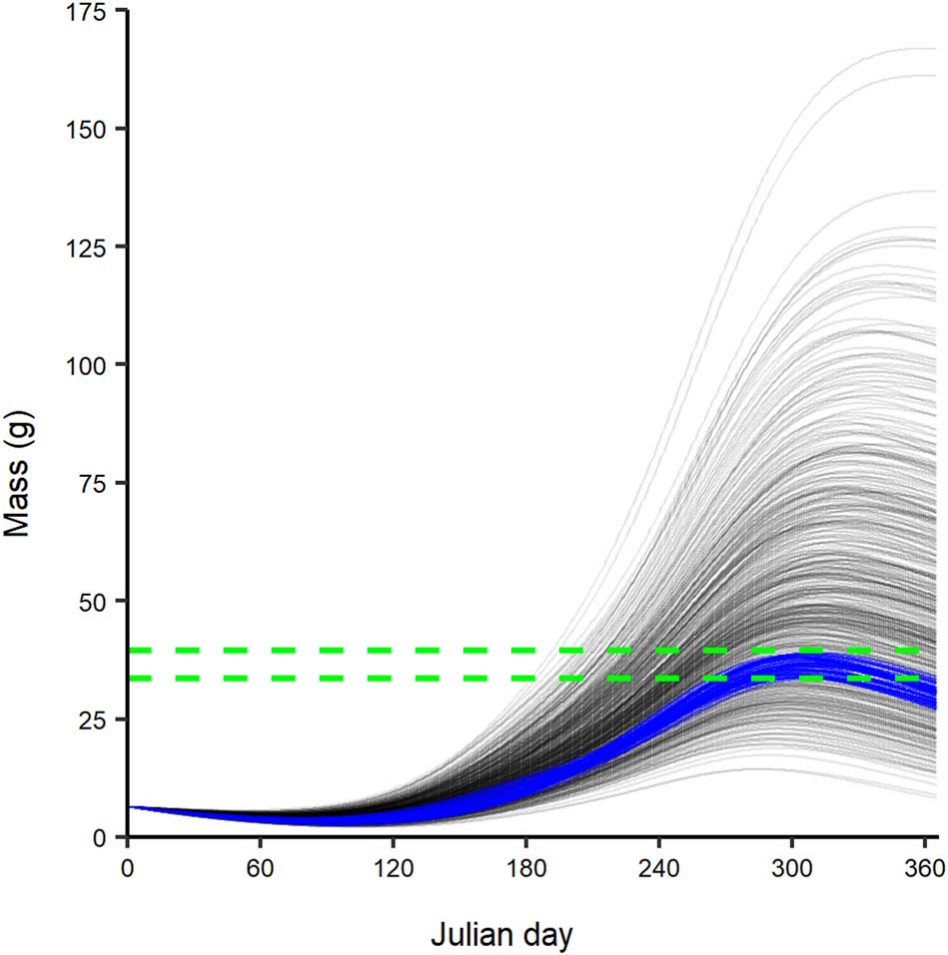
Empirical validation of Guadalupe bass BEM. Relationship between Julian day and simulated growth of age-1 Guadalupe bass in the South Llano River from 2006 to 2011. Each solid line represents 1 of 500 simulations. Blue lines (*n* = 56) represent simulated growth trajectories within ±2.5% of end-of-year empirical length (green dashed lines).

**Figure 3:**
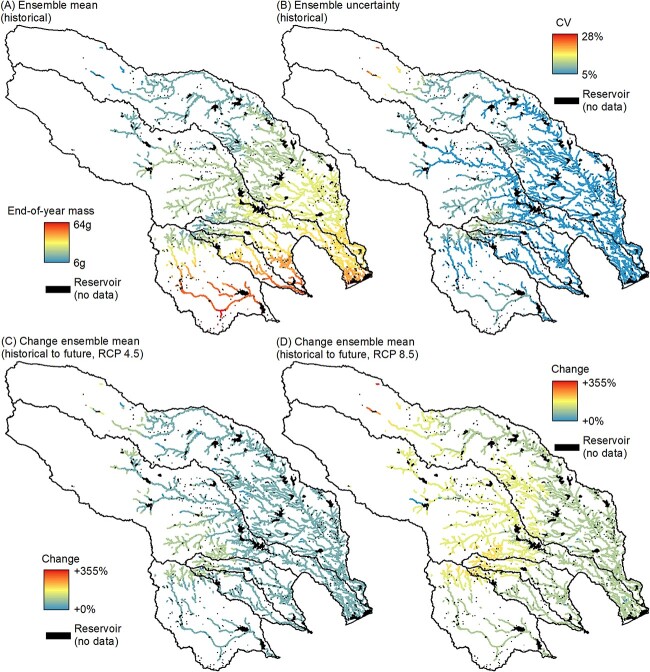
Bioenergetic model-projected performance. (**A**) Ensemble mean and (**B**) uncertainty (among 56 parameter sets) in BEM-projected growth of age-1 Guadalupe bass under historical climate for 7872 reaches within the Guadalupe bass range. Change in growth of age-1 Guadalupe bass between historical and future climate scenarios assuming (**C**) moderate and (D) high emissions scenarios.

The 500 simulated parameter sets projected a wide range of growth trajectories for age-1 Guadalupe bass; however, 56 simulations projected end-of-growing-season simulated length within ±2.5% of end-of-year empirical length in the South Llano River validation reaches (Fig. 2; Supplementary Table S2; Supplementary Fig. S3). We computed the mean (and coefficient of variation) end-of-year mass from these 56 simulations for BEM spatial interpretation. Projected end-of-year mass for age-1 fish under historical climate across 2871 historically occupied reaches was median 25.1 g (range among reaches: 16.0 g to 49.3 g). Under future climate, projected end-of-year mass increased to median 45.1 g (range among reaches: 30.0 g to 79.9 g) for the RCP 4.5 scenario and increased to median 61.5 g (range among reaches: 39.8 g to 104.3 g) for the RCP 8.5 scenario. Relative to baseline consumption (*C_P_* = 0.50), projected end-of-year mass for the RCP 4.5 scenario decreased to median 19.3 g (range among reaches: 12.1 g to 36.9 g) when consumption was suppressed (*C_P_* = 0.45) and increased to median 86.7 g (range among reaches: 59.8 g to 146.9 g) when consumption was enhanced (*C_P_* = 0.55). Across the 7872 perennial reaches, end-of-year masses were highest in the south and lowest in the north (Fig. 3A). Coefficients of variation across the 56 different simulations ranged from 5% to 28%, with greater uncertainty (higher CVs) in the north of GB range and along the southern edge of the Edwards Plateau; both areas where projected end-of-year mass was lowest (Fig. 3B). Future changes in end-of-year mass were projected to be proportionally higher for reaches in the north and on the Edwards Plateau (Fig. 3C, D). These reaches with higher future growth were in regions of historically lower temperatures that are projected to warm at a faster rate than regions with historically higher temperatures, owing to the sigmoidal shape of the Punzet *et al.* (2012) air-water temperature model (Supplementary Figs S4 and S5). Spatial patterns in end-of-year growth were similar under suppressed and enhanced consumption relative to baseline consumption (Supplementary Fig. S6).

**Figure 4:**
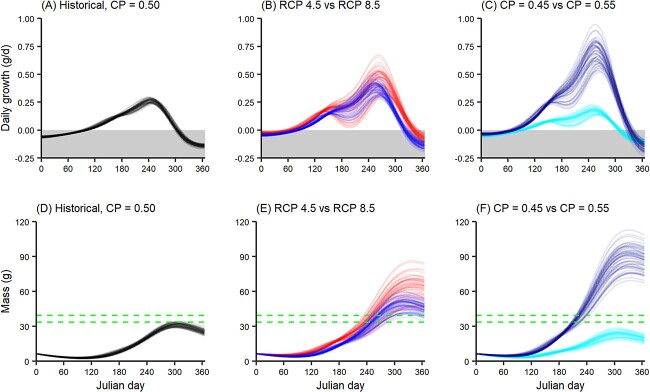
Seasonal variation in bioenergetics. Relationship between Julian day and (**A**–**C**) simulated daily growth of age-1 or (**D**–**F**) simulated cumulative growth of age-1 Guadalupe bass averaged across 2871 historically occupied reaches under (**A**, **D**) historical and (**B**, **C**) moderate (blue) versus high (red) emissions scenarios and (**E**, **F**) the moderate emissions scenario assuming suppressed consumption (*C_P_* = 0.45; light blue) versus enhanced consumption (*C_P_* = 0.55; dark blue). Each solid line represents a simulation from 1 of 56 empirically validated parameter sets. Green dashed lines correspond to ±2.5% of end-of-year empirical mass of age-1 Guadalupe bass in the South Llano River from 2006 to 2011.

Daily bioenergetic deficits were projected during the cold season and bioenergetic surpluses during the warm season under historical climate (Fig. 4A), resulting in a growing season duration of 207 days (median across 2871 historically occupied reaches; range, 194–240 days). Growing seasons were projected to increase to 243 days (range, 229–294 days) under the future RCP 4.5 scenario and 279 days (range: 258 days to 353 days) under the future RCP 8.5 scenario (Fig. 4B). Relative to baseline consumption (*C_P_* = 0.50), projected growing seasons for the RCP 4.5 scenario decreased to 220 days (range 207 days to 259 days) when consumption was suppressed (*C_P_* = 0.45) and increased to 264 days (range 247 days to 329 days) when consumption was enhanced (*C_P_* = 0.55) (Fig. 4C).

Mass was projected to increase during the growing season under historical climate, the low emissions future climate and the high emissions future climate (Fig. 4D, E). The rate of mass accumulation is more sensitive to changes in consumption relative to changes in climate (Fig. 4E, F). Specifically, the rate of mass accumulation was lower if consumption is suppressed under the low emissions future climate relative to the baseline consumption under historical climate (Fig. 4F; light blue lines). However, if consumption is enhanced, the rate of mass accumulation was higher under the low emissions future climate relative to baseline consumption under the high emissions future climate (Fig. 4F; dark blue lines).

### Congruence between bioenergetics models and species distribution models

**Figure 5:**
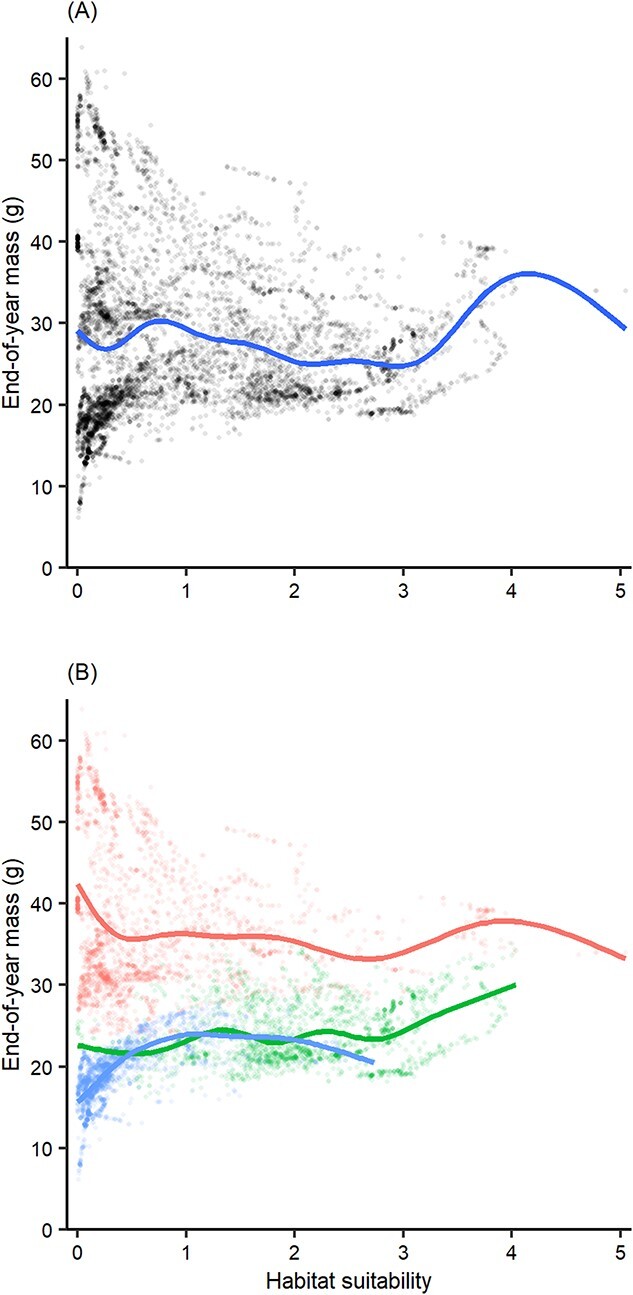
Historical concordance between BEMs and SDMs. Relationship between BEM-projected end-of-year mass and SDM-projected habitat suitability for the historical time period. Lines represent GAM fits for (**A**) all inter-confluence stream reaches and (**B**) each of the three regions: High Plains (blue), Edwards Plateau (green) and Coastal Plain (red).

End-of-year mass and habitat suitability during the historical time period were significantly correlated, but the relationship was weak and not monotonic (estimated df = 8.898, reference df = 8.997, *F* = 87.69, *P* < 0.01, deviance explained = 9.26%; Fig. 5A). Including ecoregion as a factor increased deviance explained for the model (estimated df = 8.62, reference df = 8.961, *F* = 34.34, *P* < 0.01, deviance explained = 68.0%; Fig. 5B), indicating that end-of-year mass varies more by ecoregion than by reach-specific habitat suitability. Thus, Hypothesis 1—historical end-of-year body size is positively correlated with historical habitat suitability—was not supported.

**Figure 6:**
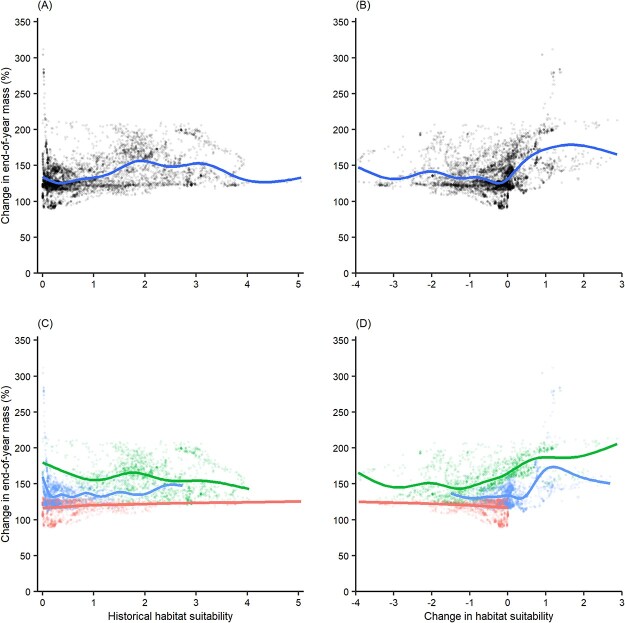
Future concordance between BEMs and SDMs. (**A**, **C**) Relationship between historical-to-future change in end-of-year mass and historical habitat suitability. (**B**, **D**) Relationship between historical-to-future change in end-of-year mass and historical-to-future change in habitat suitability. Lines represent GAM fits for (**A**, **B**) all inter-confluence stream reaches and (**C**, **D**) each of the three regions: High Plains (blue), Edwards Plateau (green) and Coastal Plain (red).

Historical to future change in end-of-year mass and historical habitat suitability were significantly correlated, but the relationship was weak and not monotonic (estimated df = 8.556, reference df = 8.947, *F* = 210.40, *P* < 0.01, deviance explained = 19.3%; Fig. 6A). Including ecoregion as a factor increased deviance explained for the model (estimated df = 8.857, reference df = 8.994, *F* = 349.5, *P* < 0.01, deviance explained = 28.6%; Fig. 6C). Thus, Hypothesis 2—historical end-of year body size is positively correlated with change in habitat suitability between historical and future time periods—was not supported.

Historical to future change in end-of-year mass and historical to future change in habitat suitability were significantly and positively correlated, indicating concordance between BEM and SDM projections (estimated df = 8.634, reference df = 8.963, *F* = 28.49, *P* < 0.01, deviance explained = 47.8%; Fig. 6B). Including ecoregion as a factor increased deviance explained for the model (estimated df = 8.619, reference df = 8.958, *F* = 300.1, *P* < 0.01, deviance explained = 59.8%; Fig. 6D). The relationship remained positive for the Edwards Plateau and High Plains (Fig. 6D, green and blue lines), but not for the Coastal Plain (Fig. 6D, red line). Thus, Hypothesis 3—change in end-of-year body size between historical and future time periods is positively correlated with change in habitat suitability between historical and future time periods—was supported.

## Discussion

### Historical distribution, ecology and physiology

A fundamental goal of ecology and an objective of this study was to elucidate drivers of geographic range limits. Geographic range limits are driven by a combination of abiotic, biotic, historical and spatial processes (Holt *et al*., 2005; Soberón, 2007). These general processes also constrain distributions of freshwater fishes, whereby drainage divides, climate, landscape geology, in-stream habitat and species interactions constrain distribution and abundance of species at broad spatial extents (Jackson *et al*., 2001; Tonn *et al*., 1990). Field studies of distribution and habitat use support this paradigm for Guadalupe bass, which associates with spring-fed streams of the Edwards Plateau (Edwards, 1980; Perkin *et al.*, 2010) and ostensibly is geographically restricted by contemporary drainage divides of the western gulf coast (Hoagstrom *et al*., 2011; Near *et al.*, 2003). Our SDM projections of historical habitat suitability confirmed the spatial association with the Edwards Plateau and the high predictive capability of baseflow index in these models confirmed the association with spring-fed streams (Curtis *et al.*, 2015). Minimum January temperature also was an important predictor of historical distribution, suggesting that *cold* winter temperatures preclude persistence in the northwestern unoccupied reaches of the Colorado and Brazos River basins. The BEM simulations under historical climate consistently projected energy deficits during the winter months and these deficits are of greater magnitude and persist for a longer duration of the cold season in the north compared to the south. These physiological projections thus support the hypothesis that cold winter temperature could limit the northern range edge of Guadalupe bass and reveals a potential mechanism of winter starvation. BEM-based studies of largemouth bass have shown that cold temperatures and short growing seasons cause starvation of juveniles and drive the cold range edge throughout North America (Fullerton *et al*., 2000; Garvey *et al*., 1998). A second (and not necessarily mutually exclusive) interpretation of high predictive capability of minimum January temperature from our SDMs is that *warm* winter temperatures preclude persistence in the southeastern unoccupied reaches along the gulf coastal plain. SDMs fitted with temperature predictors projected future erosion of habitat suitability along the southeastern range margin relative to models fitted without temperature predictors (Supplementary Fig. 2C, D), supporting the interpretation that winter temperature drives the warm range edge. Whether winter temperature actually constrains the warm-edge limit of Guadalupe bass seems dubious, given that most studies invoke intolerance to low temperature as a constrainer of cold-edge limits among ectotherms generally (Sunday *et al.*, 2011) and fishes specifically (Wood *et al.*, 2020). Nevertheless, one potential mechanism is that a cold winter—and subsequent warming in the spring—is a required seasonal spawning cue for Guadalupe bass as it is for other temperate freshwater fishes (Shuter *et al*., 2012). Our BEMs revealed explicit insight into growth and implicit insight into recruitment (e.g. size dependent winter survival; *sensu*  Fullerton *et al.*, 2000); however, additional lines of inquiry will be needed to evaluate reproduction, generally, and the seasonal cue hypothesis, specifically (Firkus *et al*., 2018; Heins, 2020). A third interpretation is that our SDMs revealed a spurious correlation between winter temperature and other important predictors—a common issue with correlative SDMs when inferring causality (Kearney and Porter, 2009). In Texas, winter and summer temperatures are not strongly correlated (Pearson *r* = −0.25), meaning that high summer temperature (a poor predictor; Supplementary Table S1) as a driver of warm-edge limits can be ruled out. From a physiological perspective, laboratory growth of Guadalupe bass indicates high optimal temperature for growth (27°C; Sullivan *et al*., 2013) relative to historical summer temperatures along the southeastern range edge (<28.5°C). Thus, it is unlikely that southern range limits are driven by sensitivity of Guadalupe bass to high summer temperatures. The northwest-to-southeast winter temperature gradient spatially correlates with the transition from Edwards Plateau to Coastal Plain and an accompanying suite of geological and biological changes (Hoeinghaus *et al*., 2007). It is likely that some combination of spuriously correlated geological and biological conditions limit Guadalupe bass populations in the Coastal Plain.

Precipitation did not provide strong predictive capability in our SDMs. This is further supported by the SDM projections under future climate scenarios that revealed similar extirpation and colonization patterns whether precipitation variables were included or not. This finding is surprising given the strong east-to-west precipitation gradient spanning the historical range of Guadalupe bass. Western range limits of Guadalupe bass are almost certainly driven by the east-to-west transition of perennial to intermittent flow regime (Muneepeerakul *et al.*, 2008; Perkin *et al.*, 2015), although our SDMs did not include explicit predictors representing flow regime. While other SDM-based studies have shown that precipitation variables can be useful surrogates of instream flow conditions (McGarvey *et al.*, 2018), this may not apply to all regions, particularly where precipitation gradients span transitions from perennial to intermittent (or ephemeral) streamflow like in Texas. Another potential explanation for poor predictive capacity of precipitation variables is that we included baseflow index—an effective surrogate for perennial flow—in our SDMs and this variable was highly predictive. The interaction between precipitation, baseflow index and the realized streamflow regime is not of the highest importance for characterizing historical range limits, but will be very important when making projections about novel future climate, flow and Guadalupe bass population viability (Birdsong *et al.*, 2015; Jiménez-Valverde *et al.*, 2009).

Competition with a more widespread congener and/or an interaction milieu associated with high-richness paratopic assemblages often limit ranges of endemic species (Graham *et al.*, 2010; Jankowski *et al.*, 2010). We did not explicitly incorporate biotic factors into our SDMs or BEMs, although prey availability and/or interspecific competition may be important drivers of Guadalupe bass range limits, as they are in other freshwater fishes (Finstad *et al.*, 2011; Taniguchi and Nakano, 2000). The eastern range limit of Guadalupe bass corresponds with a rapid increase in fish species richness (Hubbs *et al.*, 2008; Muneepeerakul *et al.*, 2008), which may intensify competition and reduce realized consumption (i.e. reduce the value of the *C_P_* parameter in our BEMs). Prey production likely also decreases along southeast-to-northwest climatic gradients (Patrick *et al.*, 2019), and similarly may reduce realized consumption of Guadalupe bass along the northern and western range limit. Although we did not extrapolate SDMs or BEMs beyond drainage basins in which Guadalupe bass historically have occurred, it is likely that BEMs (and to a lesser extent SDMs) would identify suitable habitat in drainages to the east where thermal regimes are similar (e.g. Trinity and Sabine River basins).

We conclude that historical range limits of Guadalupe bass are driven by multiple factors and that SDMs and BEMs offer different yet informative insights into these drivers. Further refinement of both modelling approaches—particularly the incorporation of (i) proximal abiotic factors (e.g. flow intermittency) and (ii) biotic factors (e.g. spatial and seasonal variation in prey density)—will improve understanding of Guadalupe bass distribution and abundance. Finally, integration with additional modelling approaches that can incorporate spatial variation in reproduction will also improve understanding of range edge dynamics of Guadalupe bass.

### Climate change impacts

A primary objective of this study was to assess vulnerability of Guadalupe bass to climate change. Our SDM-based and physiology-based assessments yielded different answers—SDMs forecasted a decline in habitat suitability, whereas BEMs forecasted an increase in growth capacity. Nevertheless, the alternative approaches yielded complementary insights into the spatial distribution of vulnerability and potential mechanisms contributing to vulnerability. Climate envelope approaches (i.e. correlative SDMs; *sensu*  Elith and Leathwick, 2009) often forecast geographic range shifts towards colder and wetter regions (Ackerly *et al.*, 2010; Comte and Grenouillet, 2013). Our SDMs matched this expected response to temperature—habitat suitability erodes at the warm-range edge in the southeastern Coastal Plain. The key environmental variable driving this forecasted erosion—minimum winter temperature—however, is an unlikely driver of warm-range edge retraction (Sunday *et al.*, 2012). Our BEMs, on the other hand, projected an increase in growth potential throughout the historical range, including along the southeastern Coastal Plain. Other physiology-based forecasts indicate similar responses in growth potential for temperate freshwater fishes (Hartman, 2017; Pease and Paukert, 2014) and other temperate ectothermic taxa (Deutsch *et al.*, 2008) under climate change. This increased growth potential is feasible, given that future water temperatures are not forecasted to exceed optimal temperature for growth (27°C; Sullivan *et al.*, 2013). However, growth potential would have to be matched by increased prey availability and while not being impeded by stronger competition with existing competitors and/or novel competition in reassembled communities (Urban *et al*., 2012). Indeed, our scenarios of suppressed and enhanced consumption demonstrated that changes in prey quantity or quality are more important than changes in temperature.

On the northern range edge, SDMs projected modest increases habitat suitability that—assuming dispersal opportunities exist—could facilitate colonization of some historically unoccupied reaches in the Colorado and Brazos River basins. BEMs also forecast the highest historical-to-future increase in growth potential in the northwest (as well as in the southwestern portion of the Edwards Plateau). This is caused by the WT model projecting the greatest increase in WT in regions with 10–20°C air temperatures (due to sigmoidal air-water relationship; Supplementary Fig. S5; Punzet *et al.*, 2012). This artefact of our simplistic air-water temperature model highlights the importance of improved understanding and forecasting of stream temperatures under anticipated changes in air temperature and surface-groundwater interactions (Caldwell *et al.*, 2020).

Surprisingly, our SDMs did not forecast an eastward shift in habitat suitability away from the dry west and towards the wet east. This is likely the result of a breakdown in the causal links between precipitation, in-stream flow conditions and Guadalupe bass occupancy. Indeed, perennial streams on which Guadalupe bass rely are forecasted to decline across historical Guadalupe bass range, particularly in the arid western portions of the Colorado and Brazos River basins (Smith *et al*., 2013). Thus, it is likely that Guadalupe bass populations on the western range edge will be lost as their habitats transition from perennial to intermittent (Perkin *et al.*, 2015). Given the contribution of the Edwards-Trinity Aquifer to surface flow of perennial streams, the extent and magnitude of dewatering will depend on amplifying interactions between climate change (i.e. as we have projected) and groundwater extraction (i.e. not explicitly included in our SDMs). We did not project habitat suitability or growth potential in the basins to the east of the Brazos River basin (i.e. Trinity and Sabine River basins) because there are no natural dispersal opportunities. Whether habitat suitability and/or growth potential would increase in these adjacent basins under future climate would likely depend on in-stream conditions (e.g. proximal correlates of baseflow index).

Another objective of our BEM assessment was to evaluate seasonal changes in Guadalupe bass growth caused by warming in the 21st century. Our BEMs indicate an energy deficit during the cold season when metabolic costs exceed consumptive gains. Warmer year-round temperatures throughout Guadalupe bass range under future climate therefore are projected to advance the onset of feeding in the spring and extend feeding in the autumn, yielding a longer growing season and larger end-of-year body size. Under historical temperature regimes, Guadalupe bass are projected to maintain positive growth even in the hottest period of summer because consumptive gains can exceed metabolic losses. For future projections, particularly the RCP 8.5 scenario, metabolic costs are projected to approach or even exceed consumptive gains for several of the parameter sets meaning that growth could be suppressed both in winter and mid-summer. These changes in seasonal energy budgets will likely affect allocation of energy to somatic growth versus reproduction and subsequent population dynamics.

### Broader implications

Mechanistic niche modelling was formalized by Kearney and Porter (2009) and has informed basic research aimed at understanding the ecological processes driving species distributions as well as applied research aimed at forecasting species vulnerability to anthropogenic environmental change (e.g. Briscoe *et al.*, 2016). Nevertheless, implementation has focused largely on terrestrial insects (e.g. Buckley *et al*., 2011), amphibians (e.g. Kearney *et al.*, 2008) and reptiles (e.g. Buckley, 2008). The BEM approach used by fisheries professionals, particularly the Wisconsin model, conceptually aligns with mechanistic niche modelling (*sensu*  Kearney and Porter, 2009), but to date is not implemented at fine spatial resolutions (i.e. individual reaches) while spanning broad spatial extents (i.e. 1000s of reaches). Our study demonstrates that this novel application of BEM is generalizable to any region or fish species. Nevertheless, several challenges currently preclude broader implementation of BEMs as tools to forecast climate change vulnerability of fishes in a spatially explicit manner.

First, a challenge of developing physiology-based conservation assessments is that physiological traits of species in need of conservation are often poorly known. For example, 39% of the 700-plus species of North American freshwater fishes are imperilled (Jelks *et al.*, 2008), yet the Wisconsin model has been parameterized for only 73 fish species worldwide (Deslauriers *et al.*, 2017), few of which are among the 270-plus imperilled species in North America. In the near term, the parameter resampling approach we used (*sensu*  Petersen *et al.*, 2008)—combined with validation from field growth studies—represents an effective strategy for rapid development of BEMs for numerous imperilled species. Indeed, data on field growth are generally available and have recently been compiled and synthesized for broad-scale meta-analyses (e.g. Rypel, 2014). In the long term, physiological reaction norms for functionally and phylogenetically underrepresented taxa should be accumulated (Chown and Gaston, 2016). Second, high spatiotemporal resolution temperature data for freshwater habitats are needed. Indeed, poorly known air–water temperature relationships and the influence of surface–groundwater interactions contributed to uncertainty in our BEM projections. Current open-source datasets tend to be spatially comprehensive, often spanning tens of thousands of reaches, but lack daily temporal resolution needed for BEM (NorWeST; Isaak *et al.*, 2017). It is likely that spatiotemporally comprehensive data will become available in the near future (e.g. Hydroclim; hydroclim.org) as the open-source data culture grows (McManamay and Utz, 2014). Third, spatiotemporally comprehensive biological information is needed to better understand how predator–prey interactions (i.e. spatiotemporal variation in *C_P_* parameter) and behaviour (i.e. spatiotemporal variation in *ACT* parameter) vary along environmental gradients and among seasons. Surrogates of these factors can be acquired from existing geospatial datasets (e.g. net primary production; Deines *et al*., 2015) or statistical models (e.g. secondary production; Patrick *et al.*, 2019). This information will facilitate the adjustment of *C_P_* and *ACT* parameters in BEMs to more realistically project energy budgets of fish.

## Conclusions

Our work demonstrates the utility of applying an ensemble of approaches to assess ecological responses to future climate change (Hollowed *et al*., 2013). Comparison between habitat suitability based on SDM and growth capacity based on BEM indicated no spatial concordance between the two approaches, whereas comparison between historical to future change in these responses revealed spatial concordance between the two approaches. The emergent pattern is that habitat suitability may or may not correlate with potential for end-of-year mass, perhaps because increases in temperature are interpreted differently between modelling approaches. In the BEM approach, warmer temperatures that do not exceed critical thermal maxima result in greater gain in mass (assuming unlimited resources for growth); whereas, in the SDM approach, correlative temperature relationships equate to occurrence based on unknown and unmeasured mechanisms. It is possible that unmet increases in energetic demand under greater temperatures is a mechanism for the modelled reduction in habitat suitability as discovered in other systems (Johansen *et al.*, 2015; Morgan *et al.*, 2001), particularly in the High Plains and Edwards Plateau regions where warming is projected to increase most. Additional laboratory research could test this hypothesis. Regardless, the message remains that inference into the effects of climate change on fish distributions depends on the methodology used in the assessment and our results point to the potential for unique and perhaps synergistic insights if BEM are integrated to a greater extent.

## FUNDING

There is no funding to report.

## DATA AVAILABILITY

Data inputs were derived from sources in the public domain. Novel data were generated using original scripts and are available on the GitHub repository: https://github.com/troiamj/GB_BEM_v01.

## Supplementary Material

supp_coac035
