# Supplementary material for: Can fisheries bioenergetics modelling refine spatially explicit assessments of climate change vulnerability?
Source: Conserv Physiol. 2022 Jul 2;10(1):coac035. doi: 10.1093/conphys/coac035 (PMC9252126; doi:10.1093/conphys/coac035)

**TITLE**

Can fisheries bioenergetics modelling refine spatially-explicit assessments of climate change vulnerability?

**AUTHORS**

Matthew J. Troia

Department of Integrative Biology, University of Texas at San Antonio, San Antonio, TX 78249

Joshuah S. Perkin

Department of Ecology and Conservation Biology, Texas A&M University, College Station, TX 77843

**CORRESPONDENCE**
Matthew J. Troia

Fax: (210) 458-5005

Phone: (608) 886-6784

Email: matthew.troia@utsa.edu

**SUPPLEMENTARY MATERIAL**

Supplementary Table 1. Environmental covariates used for species distribution models.

| Covariate | Description | Range | | | Units | Source | Importance* |
| --- | --- | --- | --- | --- | --- | --- | --- |
| TotDASqKM | Catchment area | 0 | – | 207526.3 | km^2^ | NHD | 0.00 |
| SLOPE | Reach slope | 0.0001 | – | 2.17 | % | NHD | 0.00 |
| BFI | Baseflow index | 2 | – | 61.48213 | % | StreamCat | -2.01 |
| HydrlCond | Lithological hydraulic conductivity | 0.000004 | – | 224.6375 | μm·sec^-1^ | StreamCat | -0.37 |
| CompStrgth | Lithological uniaxial compressive strength | 0.333 | – | 188.6766 | megaPascals | StreamCat | -0.51 |
| Kffact | Soil erodibility (Kf) factor | 0 | – | 0.4542416 | unitless | StreamCat | -1.09 |
| Runoff | Runoff | 0 | – | 406.6479 | mm | StreamCat | 0.97 |
| Perm | Permeability of soils | 0.44 | – | 23.43 | cm·h^-1^ | StreamCat | -0.33 |
| RckDep | Depth to bedrock of soils | 30.79 | – | 152.4 | cm | StreamCat | 0.07 |
| WtDep | Seasonal water table depth of soils | 31.044918 | – | 183.9073 | cm | StreamCat | -0.24 |
| WetIndex | Composite Topographic Index | 572.475096 | – | 1765.25 | unitless | StreamCat | -0.13 |
| PctCarbRes | Lithology: carbonate residual material | 0 | – | 100 | % | StreamCat | 0.10 |
| PctNonCarb | Lithology: non-carbonate residual material | 0 | – | 100 | % | StreamCat | 0.39 |
| PctSilicic | Lithology: silicic residual material | 0 | – | 100 | % | StreamCat | 0.04 |
| PctEolCrs | Lithology: eolian coarse sediment | 0 | – | 100 | % | StreamCat | 0.36 |
| PctEolFine | Lithology: eolian fine sediment | 0 | – | 5.048054 | % | StreamCat | 0.01 |
| PctSalLake | Lithology: saline lake sediment | 0 | – | 4.316827 | % | StreamCat | 0.00 |
| PctAlluvCo | Lithology: coarse alluvium | 0 | – | 100 | % | StreamCat | 0.42 |
| Tmax07 | Max. temp. in the warmest month | 31.9 | – | 37.4 | °C | ClimateNA | 0.06 |
| Tmin01 | Min. temp. in the coldest month | -6.7 | – | 7.4 | °C | ClimateNA | -1.13 |
| PPT05 | Prec. in the wettest month | 43 | – | 143 | mm | ClimateNA | 0.10 |
| PPT10 | Prec. in the driest month | 30 | – | 129 | mm | ClimateNA | 0.74 |
| PPT07 | Prec. in the warmest month | 30 | – | 114 | mm | ClimateNA | -0.16 |
| PPT01 | Prec. in the coldest month | 9 | – | 99 | mm | ClimateNA | 0.62 |
| * Percent change in AUC with the covariate withheld from the model. Value represents mean of 10 independent cross validations. | | | | | | | |

Supplementary Table 2. Parameter set for largemouth bass and for 56 synthetic parameter sets used to project Guadalupe bass growth.

| Parameter set | C_P_ | C_A_ | C_B_ | CTM | CTO | C_Q_ | R_ACT_ | R_A_ | R_B_ | R_Q_ | F_A_ | U_A_ | SDA | ED |
| --- | --- | --- | --- | --- | --- | --- | --- | --- | --- | --- | --- | --- | --- | --- |
| LMB^A^ | 0.5 | 0.33 | -0.325 | 37.0 | 27.5 | 2.65 | 1.0 | 0.0084 | -0.355 | 0.0313 | 0.104 | 0.0882 | 0.16 | 4184 |
| 9 | 0.5 | 0.33 | -0.319 | 38.4 | 28.3 | 2.61 | 1.0 | 0.0086 | -0.361 | 0.0324 | 0.107 | 0.0844 | 0.16 | 4119 |
| 19 | 0.5 | 0.34 | -0.337 | 37.8 | 27.9 | 2.77 | 1.0 | 0.0083 | -0.362 | 0.0327 | 0.104 | 0.0880 | 0.16 | 4254 |
| 21 | 0.5 | 0.33 | -0.315 | 38.7 | 28.5 | 2.53 | 1.0 | 0.0085 | -0.355 | 0.0316 | 0.100 | 0.0841 | 0.17 | 4373 |
| 23 | 0.5 | 0.33 | -0.330 | 37.2 | 28.2 | 2.73 | 1.0 | 0.0086 | -0.357 | 0.0302 | 0.106 | 0.0846 | 0.16 | 4183 |
| 36 | 0.5 | 0.32 | -0.320 | 36.8 | 26.8 | 2.71 | 1.0 | 0.0086 | -0.356 | 0.0323 | 0.101 | 0.0873 | 0.17 | 4394 |
| 39 | 0.5 | 0.34 | -0.331 | 38.4 | 28.2 | 2.70 | 1.0 | 0.0081 | -0.350 | 0.0326 | 0.106 | 0.0851 | 0.16 | 4305 |
| 70 | 0.5 | 0.33 | -0.312 | 35.5 | 27.8 | 2.78 | 1.0 | 0.0087 | -0.352 | 0.0328 | 0.104 | 0.0859 | 0.15 | 3982 |
| 74 | 0.5 | 0.33 | -0.319 | 36.9 | 28.4 | 2.64 | 1.0 | 0.0087 | -0.352 | 0.0313 | 0.109 | 0.0906 | 0.15 | 4013 |
| 80 | 0.5 | 0.33 | -0.317 | 35.8 | 28.4 | 2.67 | 1.0 | 0.0084 | -0.363 | 0.0326 | 0.106 | 0.0854 | 0.16 | 4279 |
| 84 | 0.5 | 0.33 | -0.314 | 38.5 | 28.1 | 2.67 | 1.0 | 0.0084 | -0.339 | 0.0324 | 0.101 | 0.0843 | 0.16 | 4145 |
| 105 | 0.5 | 0.32 | -0.328 | 38.1 | 28.1 | 2.70 | 1.0 | 0.0081 | -0.352 | 0.0298 | 0.107 | 0.0845 | 0.16 | 4353 |
| 116 | 0.5 | 0.33 | -0.319 | 36.1 | 28.5 | 2.75 | 1.0 | 0.0081 | -0.343 | 0.0319 | 0.102 | 0.0840 | 0.16 | 4184 |
| 124 | 0.5 | 0.32 | -0.337 | 37.6 | 27.9 | 2.56 | 1.0 | 0.0086 | -0.351 | 0.0306 | 0.106 | 0.0839 | 0.15 | 4035 |
| 131 | 0.5 | 0.32 | -0.337 | 36.0 | 27.7 | 2.70 | 1.0 | 0.0086 | -0.371 | 0.0302 | 0.100 | 0.0845 | 0.16 | 4130 |
| 132 | 0.5 | 0.31 | -0.325 | 35.3 | 28.3 | 2.63 | 1.0 | 0.0081 | -0.356 | 0.0307 | 0.108 | 0.0892 | 0.16 | 4117 |
| 141 | 0.5 | 0.34 | -0.339 | 35.5 | 28.1 | 2.60 | 1.0 | 0.0082 | -0.346 | 0.0321 | 0.105 | 0.0923 | 0.16 | 4144 |
| 148 | 0.5 | 0.33 | -0.313 | 38.0 | 28.1 | 2.56 | 1.0 | 0.0086 | -0.342 | 0.0307 | 0.106 | 0.0899 | 0.16 | 4109 |
| 159 | 0.5 | 0.32 | -0.309 | 38.2 | 28.8 | 2.70 | 1.0 | 0.0084 | -0.355 | 0.0307 | 0.104 | 0.0851 | 0.15 | 3986 |
| 165 | 0.5 | 0.33 | -0.323 | 38.4 | 28.6 | 2.57 | 1.0 | 0.0084 | -0.354 | 0.0328 | 0.099 | 0.0844 | 0.16 | 4288 |
| 210 | 0.5 | 0.32 | -0.323 | 35.3 | 28.1 | 2.65 | 1.0 | 0.0081 | -0.347 | 0.0309 | 0.100 | 0.0848 | 0.16 | 4345 |
| 212 | 0.5 | 0.32 | -0.326 | 37.4 | 26.3 | 2.74 | 1.0 | 0.0084 | -0.338 | 0.0325 | 0.102 | 0.0926 | 0.16 | 4144 |
| 233 | 0.5 | 0.32 | -0.339 | 35.2 | 27.0 | 2.77 | 1.0 | 0.0087 | -0.370 | 0.0319 | 0.100 | 0.0915 | 0.16 | 4103 |
| 237 | 0.5 | 0.32 | -0.313 | 38.4 | 28.7 | 2.61 | 1.0 | 0.0083 | -0.356 | 0.0315 | 0.100 | 0.0863 | 0.16 | 4208 |
| 246 | 0.5 | 0.34 | -0.336 | 36.5 | 28.6 | 2.72 | 1.0 | 0.0080 | -0.347 | 0.0309 | 0.105 | 0.0844 | 0.15 | 4021 |
| 254 | 0.5 | 0.33 | -0.336 | 38.6 | 26.7 | 2.54 | 1.0 | 0.0087 | -0.342 | 0.0325 | 0.107 | 0.0872 | 0.16 | 4109 |
| 268 | 0.5 | 0.32 | -0.323 | 35.2 | 26.7 | 2.69 | 1.0 | 0.0084 | -0.342 | 0.0321 | 0.106 | 0.0923 | 0.16 | 4166 |
| 274 | 0.5 | 0.33 | -0.321 | 36.2 | 28.2 | 2.78 | 1.0 | 0.0082 | -0.354 | 0.0299 | 0.106 | 0.0893 | 0.16 | 4319 |
| 278 | 0.5 | 0.33 | -0.322 | 37.6 | 28.1 | 2.68 | 1.0 | 0.0086 | -0.350 | 0.0320 | 0.103 | 0.0880 | 0.15 | 4019 |
| 282 | 0.5 | 0.33 | -0.316 | 37.5 | 28.1 | 2.75 | 1.0 | 0.0083 | -0.372 | 0.0327 | 0.106 | 0.0888 | 0.17 | 4368 |
| 283 | 0.5 | 0.34 | -0.325 | 35.3 | 28.2 | 2.66 | 1.0 | 0.0086 | -0.360 | 0.0323 | 0.104 | 0.0918 | 0.16 | 4252 |
| 287 | 0.5 | 0.34 | -0.341 | 37.9 | 27.2 | 2.77 | 1.0 | 0.0085 | -0.340 | 0.0322 | 0.105 | 0.0851 | 0.16 | 4104 |
| 288 | 0.5 | 0.33 | -0.337 | 35.9 | 28.3 | 2.53 | 1.0 | 0.0081 | -0.347 | 0.0305 | 0.101 | 0.0901 | 0.17 | 4372 |
| 311 | 0.5 | 0.32 | -0.327 | 36.3 | 28.5 | 2.54 | 1.0 | 0.0080 | -0.349 | 0.0312 | 0.105 | 0.0848 | 0.16 | 4160 |
| 314 | 0.5 | 0.34 | -0.326 | 36.0 | 27.0 | 2.72 | 1.0 | 0.0086 | -0.346 | 0.0322 | 0.102 | 0.0923 | 0.16 | 4333 |
| 318 | 0.5 | 0.33 | -0.321 | 37.1 | 28.5 | 2.68 | 1.0 | 0.0087 | -0.363 | 0.0318 | 0.099 | 0.0858 | 0.15 | 4011 |
| 321 | 0.5 | 0.32 | -0.337 | 38.4 | 26.6 | 2.55 | 1.0 | 0.0087 | -0.343 | 0.0305 | 0.103 | 0.0888 | 0.16 | 4202 |
| 352 | 0.5 | 0.32 | -0.329 | 36.4 | 26.4 | 2.70 | 1.0 | 0.0087 | -0.341 | 0.0308 | 0.101 | 0.0888 | 0.17 | 4365 |
| 362 | 0.5 | 0.31 | -0.313 | 38.7 | 28.7 | 2.63 | 1.0 | 0.0081 | -0.370 | 0.0314 | 0.101 | 0.0864 | 0.16 | 4305 |
| 380 | 0.5 | 0.32 | -0.312 | 37.1 | 27.9 | 2.59 | 1.0 | 0.0086 | -0.344 | 0.0306 | 0.101 | 0.0887 | 0.16 | 4231 |
| 384 | 0.5 | 0.32 | -0.324 | 35.5 | 28.2 | 2.54 | 1.0 | 0.0080 | -0.345 | 0.0307 | 0.106 | 0.0907 | 0.16 | 4250 |
| 390 | 0.5 | 0.32 | -0.326 | 37.5 | 27.2 | 2.63 | 1.0 | 0.0087 | -0.341 | 0.0307 | 0.103 | 0.0860 | 0.15 | 4016 |
| 392 | 0.5 | 0.33 | -0.335 | 35.3 | 27.9 | 2.62 | 1.0 | 0.0084 | -0.354 | 0.0320 | 0.108 | 0.0853 | 0.16 | 4116 |
| 407 | 0.5 | 0.34 | -0.325 | 37.9 | 28.6 | 2.72 | 1.0 | 0.0083 | -0.366 | 0.0319 | 0.106 | 0.0856 | 0.16 | 4195 |
| 418 | 0.5 | 0.32 | -0.340 | 35.3 | 26.6 | 2.73 | 1.0 | 0.0086 | -0.355 | 0.0309 | 0.102 | 0.0900 | 0.16 | 4101 |
| 431 | 0.5 | 0.33 | -0.312 | 37.6 | 28.5 | 2.59 | 1.0 | 0.0080 | -0.345 | 0.0328 | 0.102 | 0.0925 | 0.16 | 4204 |
| 441 | 0.5 | 0.33 | -0.321 | 38.0 | 28.7 | 2.59 | 1.0 | 0.0086 | -0.363 | 0.0310 | 0.104 | 0.0919 | 0.15 | 4057 |
| 451 | 0.5 | 0.33 | -0.341 | 37.7 | 28.6 | 2.57 | 1.0 | 0.0080 | -0.348 | 0.0307 | 0.106 | 0.0897 | 0.15 | 4052 |
| 457 | 0.5 | 0.32 | -0.328 | 37.5 | 27.7 | 2.56 | 1.0 | 0.0086 | -0.352 | 0.0325 | 0.102 | 0.0888 | 0.15 | 4047 |
| 458 | 0.5 | 0.32 | -0.325 | 35.3 | 27.6 | 2.78 | 1.0 | 0.0086 | -0.366 | 0.0300 | 0.107 | 0.0900 | 0.15 | 4067 |
| 464 | 0.5 | 0.34 | -0.325 | 36.1 | 28.8 | 2.67 | 1.0 | 0.0084 | -0.351 | 0.0308 | 0.108 | 0.0852 | 0.15 | 4088 |
| 470 | 0.5 | 0.34 | -0.333 | 37.2 | 27.6 | 2.76 | 1.0 | 0.0086 | -0.353 | 0.0324 | 0.105 | 0.0896 | 0.16 | 4292 |
| 477 | 0.5 | 0.34 | -0.316 | 38.6 | 28.8 | 2.77 | 1.0 | 0.0085 | -0.368 | 0.0308 | 0.104 | 0.0889 | 0.16 | 4285 |
| 479 | 0.5 | 0.32 | -0.340 | 38.4 | 26.6 | 2.65 | 1.0 | 0.0086 | -0.349 | 0.0309 | 0.103 | 0.0913 | 0.16 | 4189 |
| 488 | 0.5 | 0.32 | -0.335 | 35.2 | 27.2 | 2.66 | 1.0 | 0.0085 | -0.348 | 0.0299 | 0.100 | 0.0891 | 0.17 | 4383 |
| 490 | 0.5 | 0.32 | -0.333 | 37.9 | 26.4 | 2.67 | 1.0 | 0.0087 | -0.340 | 0.0302 | 0.101 | 0.0902 | 0.16 | 4305 |
| 495 | 0.5 | 0.34 | -0.327 | 36.9 | 28.6 | 2.75 | 1.0 | 0.0080 | -0.340 | 0.0310 | 0.100 | 0.0904 | 0.15 | 4063 |
| ^A^ Largemouth bass from Rice et al. 1983 | | | | | | | | | | | | | | |

Supplementary Figure 1. Delineation of High Plains (EPA Level III codes 25, 26, 27, 29), Edwards Plateau (EPA Level III code 30), and Coastal Plain (EPA Level III codes 31, 32, 33, 34, 35) regions.


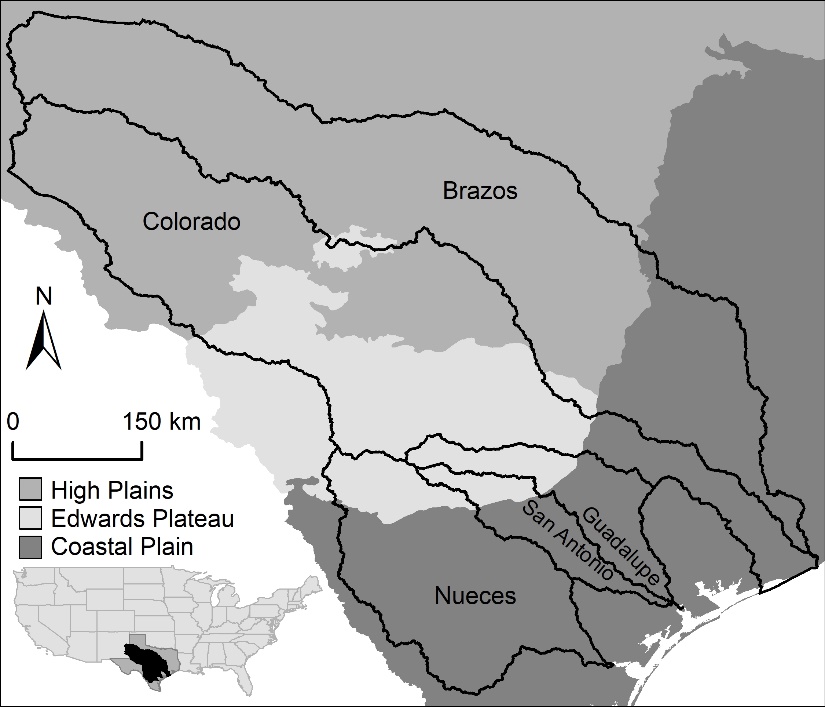


Supplementary Figure 2. SDM-projected occupancy transitions between historical and future climate scenarios assuming (A,C) moderate and (B,D) high emissions scenarios. Projections assume (A-B) altered temperature only and (C-D) altered precipitation only. Panels A, B, C, and D correspond to projections 3, 6, 4, and 7, respectively, from Table 1. See Figure 1 for altered temperature and precipitation. Pie charts show proportion of 7,872 reaches in each occupancy transition. Panels (A), (B), (C), and (D) illustrate projections 3, 6, 4, and 7 from Table 1, respectively.


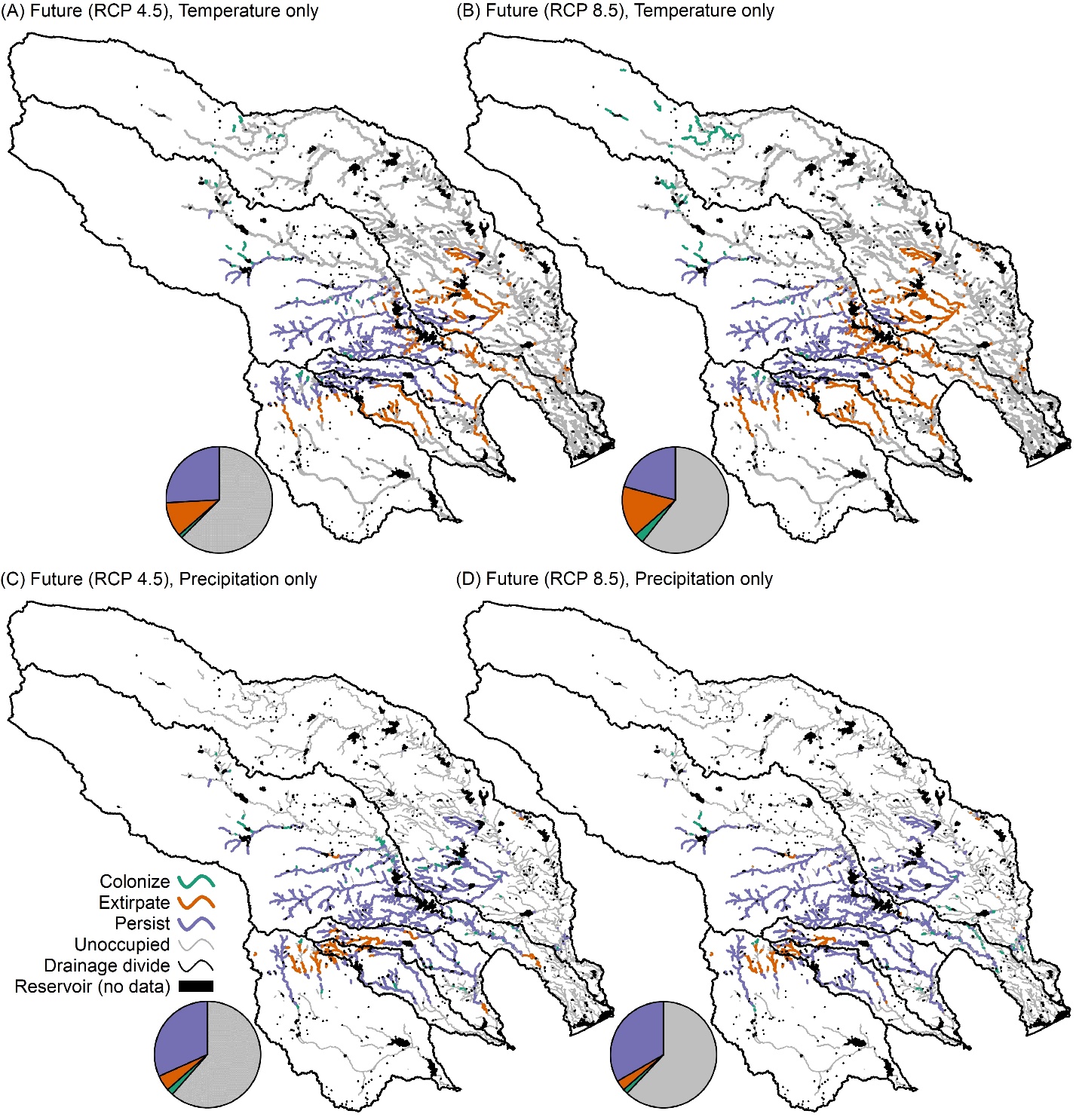


Supplementary Figure 3. Relationships between (A) respiration and temperature, (B) respiration and mass, (C) consumption and temperature, and (D) consumption and mass. Black lines represents each of 500 parameter sets. Red lines represent 56 parameters sets retained for projections.


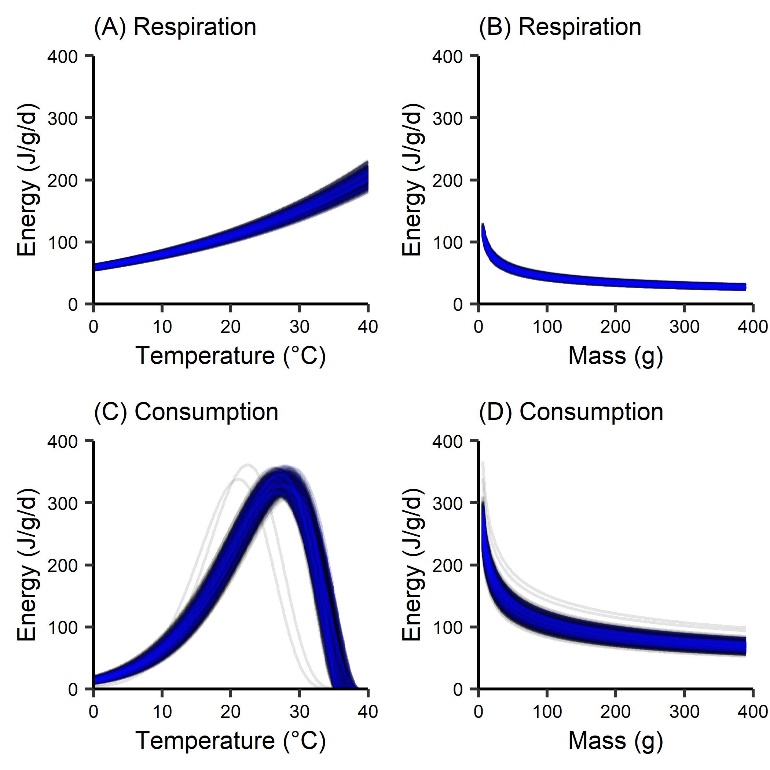


Supplementary Figure 4. Relationships between mean daily air and water temperature and days since March 1st under historical and future climate scenarios. Daily temperature (air temperature = AT, water temperature = WT) values are interpolated from mean monthly temperatures. Solid lines are the median values across 7,872 reaches and confidence bands span the minima and maxima.

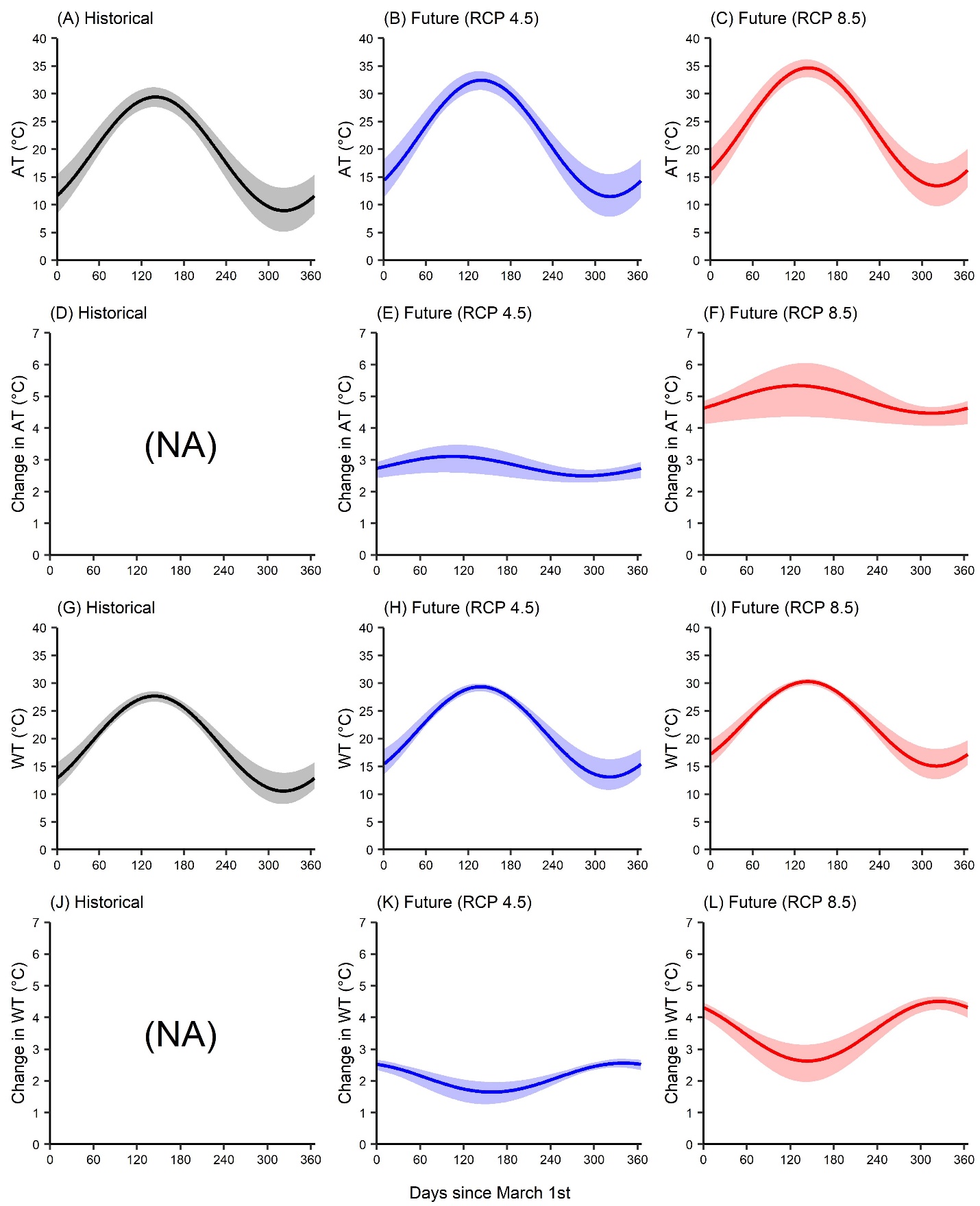


Supplementary Figure 5. Sigmoidal air-water temperature relationships for the warm-temperate Köppen–Geiger climate zone. Each point is the temperature of a reach (*n* = 7,872) under historical climate (black) and future climate assuming moderate (blue) and high (red) emissions.

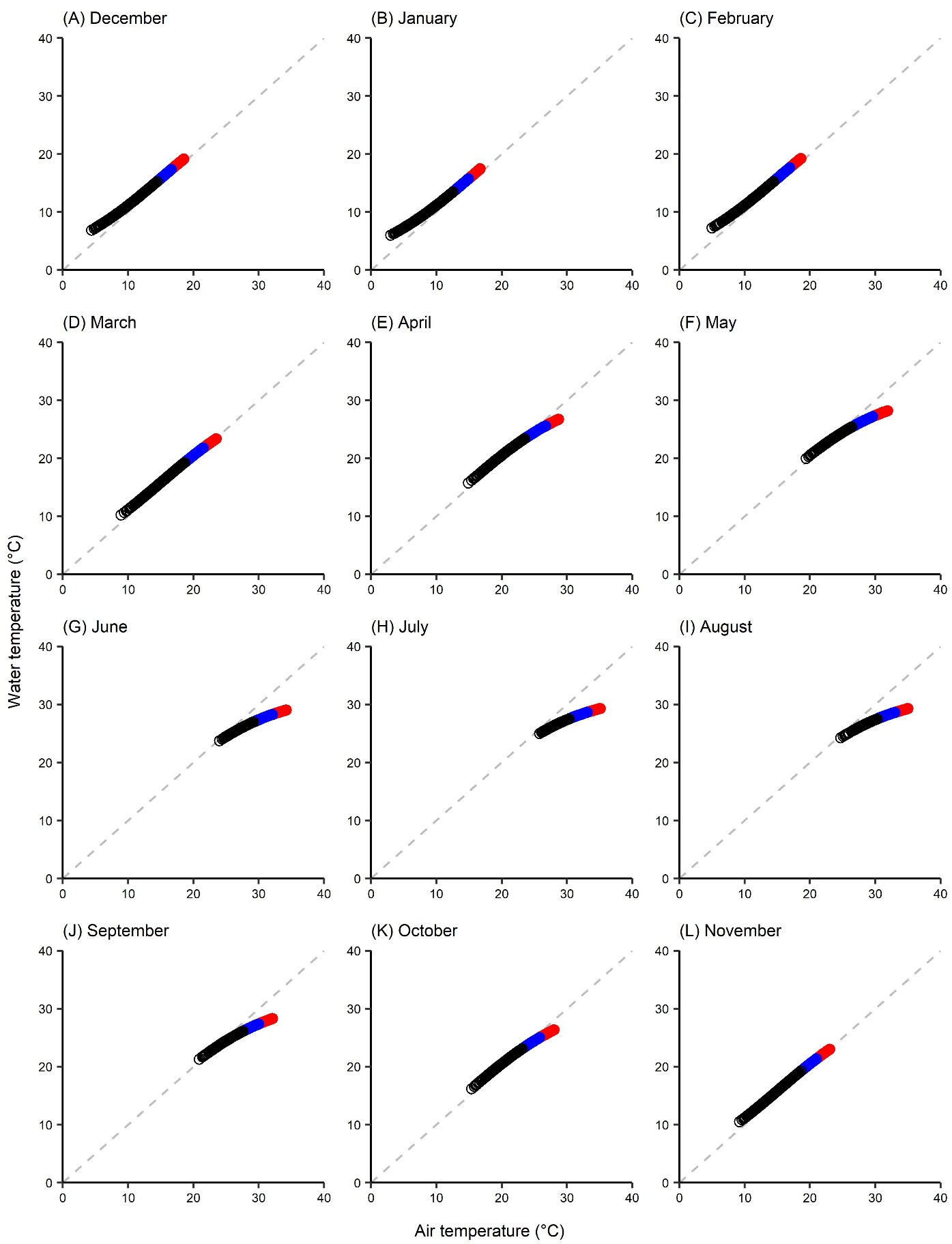


Supplementary Figure 6. (A) Ensemble mean and (B) uncertainty (among 56 parameter sets) in BEM-projected growth of age-1 Guadalupe bass for 7,872 reaches within the Guadalupe bass range under the future moderate emissions scenario assuming (A) baseline consumption (*C_P_* = 0.45), (B) suppressed consumption (*C_P_* = 0.45), or (C) enhanced consumption (*C_P_* = 0.55).


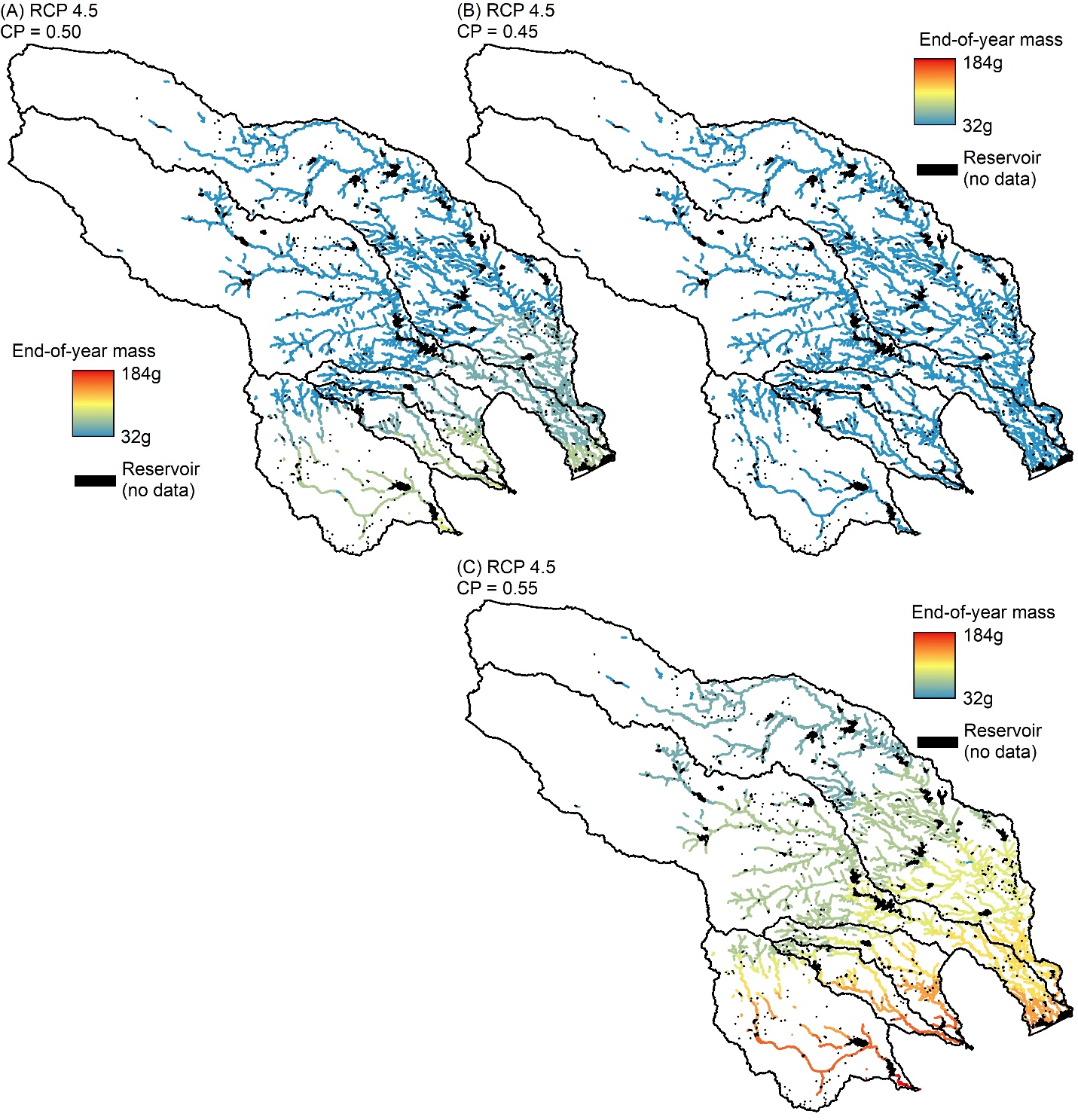

Supplement: supp_coac035 [file supp_coac035.zip › manuscript_v10_SI.docx]
